# A Pilot Study Comparing a Community of Practice Group Therapy Program with and without Concurrent Ketamine-assisted Therapy

**DOI:** 10.1192/j.eurpsy.2023.269

**Published:** 2023-07-19

**Authors:** V. W. L. Tsang, R. Moyer, P. Kryskow, Z. Walsh, S. Dames

**Affiliations:** 1 Psychiatry; 2 Interdisciplinary Studies Graduate Program, UBC, Vancouver; 3 Health and Human Services, Vancouver Island University, Nanaimo; 4 Department of Psychology, UBC, Kelowna, Canada

## Abstract

**Introduction:**

Healthcare practitioners (HCPs) are facing a mental health crisis. Group therapies have long been used to treat symptoms associated with PTSD, anxiety and/or depression, however no studies have investigated the role of implementing group therapy with and without ketamine-assisted therapies (KaT).

**Objectives:**

The current study investigated the effects of the Roots to Thrive (RTT) group therapy intervention both with and without adjunctive KaT.

**Methods:**

In the present study we conduct a secondary analysis of data derived from the 12-week group psychotherapy program to that of the same program with adjunct KaT. Participants were administered a series of validated psychiatric assessment tools before and after the 12-weeks. Inclusion criteria included a diagnosis of treatment resistant mental health condition (depression, PTSD and/or generalized anxiety disorder) and a score of 15 or greater on the PTSD Checklist for DSM-5 (PCL-5). To assess the effects of time x group interaction and calculate differences between the RTT only and RTT-KaT subgroups, a repeated measures ANOVA was conducted. Effect sizes were calculated through partial eta-squared.

**Results:**

Forty-nine HCPs with treatment-resistant PTSD, anxiety and/or depression were treated with the RTT group therapy model to target their symptoms. A total of 49 individuals (34 female, 10 male, 3 other) with a median age of 47 years old (SD 14.19) participated in the study. There were no statistically significant differences between RTT only (n=14) and RTT KaT (n=35) subgroups across gender [X2 (1, N=44) = 2.84, ns] or age [F (1, 36) = .257, p = .615]. From pre- to post-treatment, all patients showed significant reductions in scores of PTSD (from 39.3 to 20.99), depression (from 15.5 to 7.7) and anxiety (from 15.5 to 6.2). Two-way repeated measures ANOVA did not reveal any significant between-group differences between the RTT and RTT-KaT subgroups.

**Conclusions:**

This observational study provides preliminary support for the potential of the RTT community of care model of group therapy and adds to a small but growing body of knowledge on the integration of group therapy and the broad category of psychedelic psychotherapies. Given the rapid proliferation and expansion of KaT clinics throughout North America, the finding that KaT did not appear to impact changes related to the RTT intervention suggests the need for further research to better explain the potential impacts of relational transference between the two groups, and the distinct contributions of ketamine administration in a group therapy context.

**Disclosure of Interest:**

None Declared

